# Nanocomposite Polymer Electrolytes of Sodium Alginate and Montmorillonite Clay

**DOI:** 10.3390/molecules26082139

**Published:** 2021-04-08

**Authors:** Franciani C. Sentanin, Willian R. Caliman, Rodrigo C. Sabadini, Carla C. S. Cavalheiro, Rui F. P. Pereira, Maria M. Silva, Agnieszka Pawlicka

**Affiliations:** 1 IQSC, Universidade de São Paulo, Av. Trabalhador Sãocarlense 400, 13566-590 São Carlos-SP, Brazil; fransentanin@yahoo.com.br (F.C.S.); willian_caliman@usp.br (W.R.C.); rodoxxx@gmail.com (R.C.S.); carla@iqsc.usp.br (C.C.S.C.); 2 Centro de Química e Departamento de Química, Universidade do Minho, Gualtar, 4710-057 Braga, Portugal; rpereira@quimica.uminho.pt (R.F.P.P.); nini@quimica.uminho.pt (M.M.S.)

**Keywords:** nanocomposites, polymer electrolytes, clay, sodium alginate

## Abstract

Nanocomposite polymer electrolytes (NPEs) were synthesized using sodium alginate (Alg) and either sodium (SCa-3-Na^+^)- or lithium (SCa-3-Li^+^)-modified montmorillonite clays. The samples were characterized by structural, optical, and electrical properties. SCa-3-Na^+^ and SCa-3-Li^+^ clays’ X-ray structural analyses revealed peaks at 2θ = 7.2° and 6.7° that corresponded to the interlamellar distances of 12.3 and 12.8 Å, respectively. Alg-based NPEs X-ray diffractograms showed exfoliated structures for samples with low clay percentages. The increase of clay content promoted the formation of intercalated structures. Electrochemical Impedance Spectroscopy revealed that Alg-based NPEs with 5 wt% of SCa-3-Na^+^ clay presented the highest conductivity of 1.96 × 10^−2^ S/cm^2^, and Alg with 10 wt% of SCa-3-Li^+^ showed conductivity of 1.30 × 10^−2^ S/cm^2^, both measured at 70 °C. From UV-Vis spectroscopy, it was possible to infer that increasing concentration of clay promoted a decrease of the samples’ transmittance and, consequently, an increase of their reflectance.

## 1. Introduction

The 1960–1970s oil crisis significantly increased polymer materials prices, so, aiming to lower these materials prices, the industry turned toward substituting part of the polymer with low-cost mineral fillers as additives [1]. This necessity has attracted interest in the technical use of those mineral fillers, leading to major developments in this area. It was proved that those fillers provide a flame delay, heterogeneous nucleation, color thixotropy, lubrication, and improvement of thermal and electrical properties [2]. They are solid and nonsoluble and are used to modify the macroscopic properties, such as modulus and toughness, of the polymers [3]. Some examples of such fillers are glass and carbon fibers, pigments, and mineral silicates, among others.

Clays are defined as natural, earthy, fine-grained materials, which present plasticity when wet [4]. They are widely used as an important raw material for agriculture and industry, because of their swelling, adsorption, rheological, and colloidal properties. Therefore, they are widely used in ceramics, oil, paper, and metallurgy industries [4]. The clays’ organophilic nature reduces the surface energy, making them compatible with organic polymers. Aside from that, their basal spacing facilitates polymers’ chains intercalation between the layers. The advantage of nanocomposites over conventional composites is the low concentration of fillers [5]. For example, the addition of 3–5 wt% of clay, which corresponds to 30–50 wt% of micrometric fillers, can improve the mechanical and thermal properties of the polymer material [6]. The addition of organophilic clays to polymers results in nanocomposites with improved properties, such as toughness, hardness, and thermal stability [7]. Moreover, in polymer-clay nanocomposites, the lamellae of clays provide a structural reinforcement, which is strongly attached to the polymer. This combination optimizes physical–chemical properties, such as resistance to mechanical stresses [8], thermal stability [9], gas [10], and ultraviolet barrier [11]. It also improves the ionic conductivity, porosity, and electrolyte uptake, as already shown for polymer electrolyte membranes (PEMs) made of the mixture of poly(vinylidene fluoride) (PVDF), poly(vinylpyrrolidone) (PVP), and montmorillonite (MMT) clay, soaked in LiPF_6_ [12].

There are several ways to modify clays, including ion exchange with inorganic cations, adsorption, graphitization, delamination, dehydroxylation, and reaggregation of smectic mineral clays. There are also physical proceedings, such as lyophilization, plasma, ultrasound, and others [13]. The ion exchange technique was used in this work, which basically consists of dispersing the clay in water, adding the previously dissolved salt, and maintaining this solution under stirring for a certain period. Then, the excess of unreacted salt is removed, and the remaining precipitate is filtered, dried, and disaggregated [14].

Alginate, which is a calcium, magnesium, sodium, or potassium salt, and its derivatives are obtained from alginic acid (Halg), a linear polysaccharide that consists of monomeric units of d-mannuronic and l-guluronic acids. It is found in the wall cells of brown algae, which is the main source of alginates; it comes from *Phaeophyceae* family [15]. Alginates have been used in many industrial sectors and can be prepared by replacing their H^+^ ions with mono or divalent cations, resulting in salts.

Among different salts used as charge carriers in polymer electrolytes, the most used are those that release lithium ions [16]. For example, sodium alginate-based polymer electrolytes containing 15 wt% LiClO_4_ have shown an ionic conductivity value of 3.1 × 10^−4^ S/cm^2^ at room temperature [15], which already meets the ionic conductivity requirements of more than 10^−4^ S/cm [17,18]. Therefore, the nanocomposites based on natural polymers and modified clays can have increased conductivity properties, because of the additional Li^+^ ions’ presence. On the other hand, the increase in conductivity of nanocomposite polymer electrolytes (NPEs) can be promoted by the increase of the space in between the clay layers and insulation of the charge carriers—cations—from the negatively charged silicate sheets by the intercalated polymer [19]. In the present work, we contribute to finding the conductivity increase origin. Montmorillonite clays (SCa-3-Na^+^) were modified by the ion exchange method to produce SCa-3-Li^+^ clays. These clays were used to modify the sodium alginate polymer matrix, aiming to see how it influences the ionic conductivity of these new nanocomposite polymer electrolytes.

## 2. Results and Discussion

**Figure 1:**
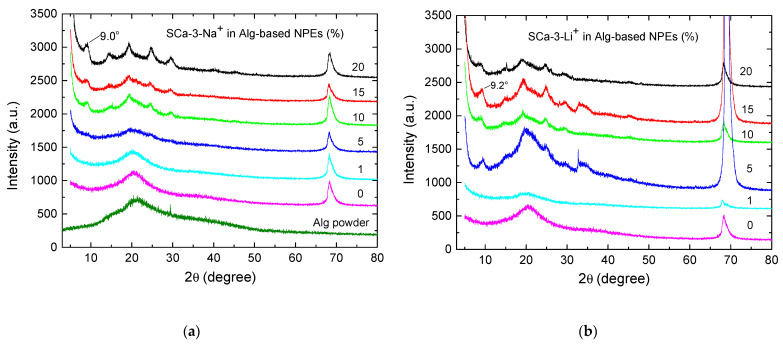
XRD for alginate powder, SCa-3-Na^+^, SCa-3-Li^+^ clays, and Alg-based NPEs with 0–20 wt% of (**a**) SCa-3-Na^+^ and (**b**) SCa-3-Li^+^.

The samples of nanocomposite membranes are composed of alginate as polymer matrix, montmorillonite clay as filler, glycerol as plasticizer, lithium perchlorate as salt, and formaldehyde. The samples were then characterized by their structural, optical, and electrical properties to evaluate clay influence on the optical and electrical behavior of these new NPEs. Therefore, X-ray diffraction was used to analyze clay structure in Alg-based NPEs. Figure 1 shows XRD patterns of alginate powder (Figure 1a) and their corresponding NPEs (0–20 wt%). As previously published, SCa-3-Na^+^ and SCa-3-Li^+^ presented peaks at 2θ = 7.2 and 6.7°, corresponding to plan (001) and interlamellar spacings of 12.3 and 12.8 Å, respectively [20]. The difference in the values between the two clays was probably due to the efficient cationic exchange [20,21] and better polymer-clay interaction, leading to an increase in interlamellar distance, seen as a shift of d(001) plane characteristic peak to a smaller angle. Alginate powder and Alg-based NPEs with no clay have only one large band centered at 2θ = 20.5°, indicating their semicrystalline structures [22]. The peak at 2θ = 70.0° comes from the silicon wafer used as support for the membranes. The addition of up to 1 wt% of clay did not make big changes. At this point, it should be mentioned that the polymer penetration can increase the distance between clay layers beyond a limit value, which results in d(001) peak disappearance. This was observed in the present study, where no d(001) peaks below 2θ = 10° were seen for the samples with 1 wt% of SCa-3-Li^+^ and 5 wt% of SCa-3-Na^+^, suggesting their exfoliated structures [21]. This behavior was already observed in NPEs of gelatin loaded with 1–15 wt% of SCa-3-Na^+^(Li^+^) [20]. At 5 wt% of SCa-3-Li^+^ (Figure 1b) and at 10 wt% of SCa-3-Na^+^ (Figure 1a), small peaks start to emerge, in the 2θ = 9–40° range, over this large band centered at 20.5°. These peaks are clearly due to the interaction of alginate with clay. The characteristic d(001) peaks at 9.0 (Figure 1a) and 9.2° (Figure 1b) correspond to 9.8 and 9.6 Å interlamellar spacings, and they point to intercalated structures, where the polymer chains enter in between clay lamellae. While these crystalline peaks did not change much for Alg-based NPEs with SCa-3-Li^+^, the NPE sample with 20 wt% of SCa-3-Na^+^ developed four peaks in 2θ = 10–35° range. Therefore, Alg-based NPEs with SCa-3-Na^+^(Li^+^) have shown a change from exfoliated to intercalated structures with the increase of the quantity of clay, which resembles the samples of gelatin-based NPEs with montmorillonite [20] and poly(methylmethacrylate) (PMMA)/MMT [23].

**Figure 2:**
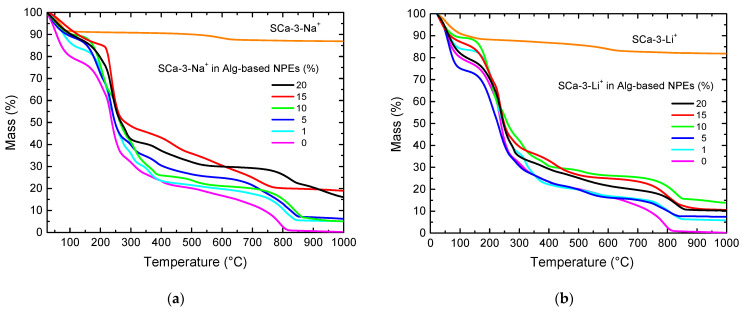
Thermograms for SCa-3-Na^+^ and SCa-3-Li^+^ clays and Alg-based NPEs with 0–20 wt% of (**a**) SCa-3-Na^+^ and (**b**) SCa-3-Li^+^.

Figure 2 shows the clays and Alg-based NPEs with 0–20% of SCa-3-Na^+^ or SCa-3-Li^+^ mass loss thermograms as a function of the temperature. It is well-known that the presence of clay in the polymer matrix improves its thermal stability, because it acts as an oxygen barrier and obstructs the exit of produced volatiles during thermal decomposition [7,24]. Both clay samples showed the same behavior, where an initial mass loss of 8% was observed from room temperature to 135 °C, presumably due to the surface and interlayer adsorbed water loss. An increase of temperature promoted one more mass loss of ~10% in the temperature range from 544 to 649 °C, with a result of clay structural -OH groups loss [7]. At 1000 °C, the total mass loss was 18%, and the remainder of the analyzed samples was probably composed of silicates and aluminates, a typical result of montmorillonite thermal treatment.

Alginate-based NPEs are mainly composed of natural polymer and clay, and their thermal stability is much lower, when compared to pure clays. Alg-based NPEs with SCa-3-Na^+^ started losing their mass at 30 °C, and this mass loss was different for different samples. Alginate-based polymer electrolytes without clay have relatively high thermal stability losing 10–20% of their mass up to 170 °C [15]. Alg-based NPEs with 0% clay displayed typical three-stage decomposition, where the first one occurred up to 170 °C accompanied by the mass loss of almost 20% and due to the residual and adsorbed water. The second mass loss was associated to the start of samples’ main degradation process, occurring in the range of 170–300 °C and accompanied by a 60% mass loss. It is likely that, in this temperature range, polymeric chains started breaking out, catalyzed by LiClO_4_, which resulted in low molecular mass segments and loss of volatiles [15]. During the third mass loss of 16%, from 300 to ~700 °C, the degradation process continued, and, at this temperature range, glycerol degradation started with liberation of volatiles and clay -OH structural groups. The fourth mass loss of 12% occurred from 700 to 850 °C. It was due to complete degradation of the samples and the volatilization of its products. Above 850 °C, there was almost no residue, and at 1000 °C, the mass residue of Alg-based NPEs with 0% of clay was practically zero.

Alg-based NPEs with 1–20% of clay behaved under thermal analysis like the sample without clay, except for less mass lost during the heating. They lost up to 16% of their mass up to 170 °C. The exceptions were the samples of Alg-based NPEs with 0, 5, and 20 wt% of SCa-3-Li^+^, with mass losses of 26, 24, and 30%, respectively. Among all the samples of Alg-based NPEs, the better thermal stability up to 170 °C was observed for the samples with 15 wt% of SCa-3Na^+^ (Figure 2a) and 10 wt% of SCa-3-Li^+^ (Figure 2b). At 1000 °C, there remained only ~5% of the mass of Alg-based NPEs with 1–10 wt% of SCa-3-Na^+^ and 16 and 19% of Alg-based NPEs with 15–20 wt% of SCa-3-Na^+^, clearly due to higher clay percentage in the NPEs formulation. Alg-based NPE with SCa-3-Li^+^ 10 wt% was more stable up to 170 °C, and, at 1000 °C, there were found 14% of ashes. These small differences between the Na^+^- and Li^+^-modified clays can be a reason for the thermal behavior and water adsorption. Overall, the thermal stability of the samples was better after the addition of clay to alginate-based polymer electrolytes, confirming the previous findings [7,24].

The new Alg-based NPEs with 0–20 wt% SCa-3-Na^+^(Li^+^) were analyzed by ATR–FTIR spectroscopy. The spectra, shown in Figure S1 (Supplementary Materials), are very similar to sodium alginate [25]. The characteristic peaks of alginate uronic acid functional groups were detected at 924 cm^−1^ and mannuronic acid at around 852 cm^−1^ [22]. The montmorillonite clay characteristic bands are in the same range of wavenumbers, where the peak at 993 cm^−1^ corresponds to Si-O-Si stretching, at 912 cm^−1^ to Al-Al-OH deformation, and at 617 cm^−1^ to coupled Al-O and Si-O out of plane [20]. Therefore, probably the montmorillonite peaks are overlapped with alginate. The more detailed analysis also revealed that band at 1032 cm^−1^, which was assigned to O-H bending [22] or C-O-C stretching [26], shifted to 1028 cm^−1^ for NPEs with 20 wt% of SCa-3-Na^+^ (Figure S2c) and to 1024 cm^−1^ for NPEs with SCa-3-Li^+^ (Figure S3c). This shift could be due to the presence of clay or measurement error.

**Figure 3:**
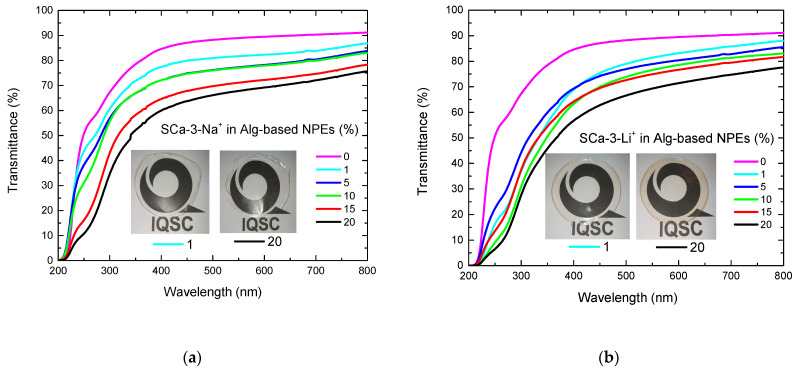
Transmittance spectra for Alg-based NPEs with 0–20 wt% of (**a**) SCa-3-Na^+^ and (**b**) SCa-3-Li^+^. Inset photos of Alg-based NPEs with 1 and 20 wt% of clay.

Solid polymer electrolytes and NPEs have been developed to substitute liquid electrolytes in electrochemical devices. One of this research’s challenges is to fulfill the visible light transparency requirement, essential for some devices. For example, transparency and/or reflectance are needed for smart electrochromic devices, especially those applied as building glazing, eyeglasses, or car windows [27]. Usually, solid electrolytes do not fulfill this requirement; however, some solid polymer electrolytes, including natural polymer electrolytes, can form thin and highly transparent films [28]. Therefore, synthesized Alg-based NPEs with SCa-3-Na^+^ or SCa-3-Li^+^ were characterized using UV–Visible light transmittance (Figure 3). All the spectra from Figure 3a reveal the same behavior, where, regardless the sample, the transmittance starts increasing from zero at 220 nm to a maximum of 75–92% at 800 nm. On the other hand, it is possible to observe a gradual decrease in transmittance with an increase in SCa-3-Na^+^ concentration. NPE with 20 wt% of clay presented 69% of transmittance at 600 nm, while NPE with 5 wt% of clay was 78% transparent. As a comparison, the Alg-based electrolyte with 0 wt% of clay had 89% of transmittance at 600 nm. These measures were done qualitatively, because the thicknesses of the samples varied between 0.04–0.10 mm (Table 1), even though we can affirm that the addition of clay decreased samples’ transparency [20].

Figure 3b shows the transmittance plots of NPEs with SCa-3-Li^+^, where the values decrease over a 200–800 nm range, with a clay content increase. NPE with 20 wt% of clay had 71% transmittance at 600 nm, while that with 5 wt% of clay had 80%. Similar results were already expected, i.e., a decrease in optical clarity with increasing clay content attributed to clay dispersion layers in the polymer matrix [29]. Therefore, the nanoscale dispersion of clay leads to exfoliated nanocomposites with lower optical clarity when compared to sample without or 1 wt% clay. This also can be seen in pictures of Alg-based NPEs membranes with 1 and 20 wt% of SCa-3-Na^+^ (Figure 3a inset) and SCa-3-Li^+^ (Figure 3b inset) clays.

**Figure 4:**
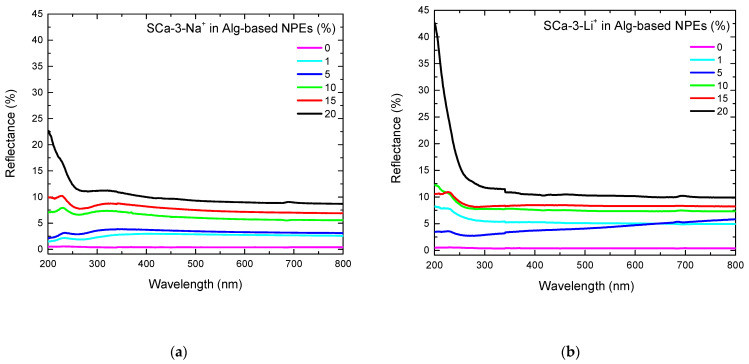
Reflectance spectra for Alg-based NPEs with 0–20 wt% of (**a**) SCa-3-Na^+^ and (**b**) SCa-3-Li^+^.

Figure 4 presents the UV-Visible reflectance spectra of Alg-based NPEs with no and two different clays. In contrast to the transmittance results, the samples’ reflectance rose with the clay content. A maximum of 11% at 320 nm was attained for the sample with 20 wt% of SCa-3-Na^+^ (Figure 4a). The same value was obtained for the sample with 20 wt% of SCa-3-Li^+^ (Figure 4b). These results are consistent with those shown in Figure 3.

**Figure 5:**
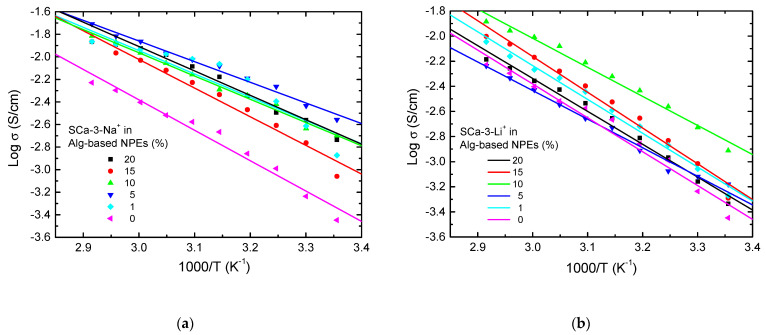
Log of conductivity in function of temperature for Alg-based NPEs with 0–20 wt% of (**a**) SCa-3-Na^+^ and (**b**) SCa-3-Li^+^.

Beyond transparency, electrical conductivity is essential for successful electrolyte use. Lu et al. [18] and Leones et al. [17] have claimed that electrolytes should satisfy requirements, such as environmental stability, electrochemical window over 1 V, low volatility, ion mobility higher than 10^−14^ m^2^/Vs, and ionic conductivity over 10^−4^ S/cm. Therefore, the NPEs samples were subjected to impedance measurements as a function of temperature. The linear increase of conductivity values, shown in Figure 5, suggested a hopping charge movement, described by the Arrhenius model [30,31]. However, some differences were observed between Alg-based NPEs with SCa-3Na^+^ (Figure 5a) and SCa-Li^+^ (Figure 5b). It seems that the hopping mechanism was predominant in Alg-based NPEs with SCa-3-Li^+^, while Alg-based NPEs with SCa-3-Na^+^ presented a mixed hopping and vehicle mechanism. Vehicular mechanism of ion transport was observed in the alginate–LiClO_4_ sample [15]; however, it was already shown that both mechanisms contributed to the conductivity in natural polymer-based electrolytes [32]. Since the charge’s movement could be described by the Arrhenius law, it was possible to calculate activation energy (Ea) from the slopes of log(σ) versus 1000/T [33].

Table 2 lists the values of ionic conductivities, at 25 and 70 °C, and activation energies (Ea) of all tested samples. Simple analysis of these results reveals an overall improvement in NPEs conductivity compared to Alg-based sample without clay. This result is similar to that observed for PEO/LiClO_4_/MMT electrolyte [34]. The Alg-based sample without clay had ionic conductivity of 3.57 × 10^−4^ S/cm^2^ at 25 °C and 5.90 × 10^−3^ S/cm^2^ at 70 °C, which is comparable to 3.1 × 10^−4^ S/cm^2^ at room temperature and 1.2 × 10^−3^ S/cm^2^ at 80 °C obtained for the sodium alginate electrolyte containing 15 wt% LiClO_4_ [15]. From all NPEs samples, the one with 5 wt% of SCa-3-Na^+^ presented highest conductivities of 2.77 × 10^−3^ S/cm^2^ at 25 °C and 1.96 × 10^−2^ S/cm^2^ at 70 °C. The highest conductivities of NPEs with SCa-3-Li^+^ were 1.22 × 10^−3^ S/cm^2^ at 25 °C and 1.30 × 10^−2^ S/cm^2^ at 70 °C for the membrane with 10 wt% SCa-3-Li^+^. A comparison of the samples has revealed that NPEs with SCa-3-Na^+^ had higher conductivities than NPEs with SCa-3-Li^+^ clays, indicating that additional Li^+^, from clay, does not improve the ionic conductivity values of the samples. Overall, both NPE samples displayed one order higher ionic conductivity value, when compared to the sample without clay, which proves that the addition of clay had a positive impact. The results presented in this paper were also better than 3.64 × 10^−6^ and 1.83 × 10^−4^ S/cm^2^ obtained for pure PVA/NaAlg and PVA/NaAlg with 10 wt% LiClO_4_, respectively [35]. Moreover, these ionic conductivity values were better than 8.0 × 10^−4^ S/cm^2^, reported by Meneghetti and Qutubuddin [36] for poly(methylmethacrylate) (PMMA) with 1.5 wt% montmorillonite clay.

From linear regression of ionic conductivity results shown in Figure 5, it was possible to calculate the activation energy for conductivity process. As expected from conductivity results, the activation energy was low and decreased gradually with an increase in clay concentration for both types of samples, i.e., NPEs containing Na^+^ and Li^+^ modified montmorillonite clays. It means that the number of ions increased with the increase of clay in the samples. Therefore, the energy barrier to the ions transport decreased, leading to decrease in the activation energy [37].

## 3. Materials and Methods

Sodium montmorillonite clay (SCa-3-Na^+^) was supplied by Otay San Diego and purified [20]. Then, SCa-3-Na^+^ clay was modified to SCa-3-Li^+^, following the ion exchange method. A total of 10.0 g of SCa-3-Na^+^ was dispersed in 500 mL of deionized water and stirred constantly for 24 h. Then, 0.32 g of LiCl was added and stirred for 48 h. After that, the obtained solution was centrifuged for 30 min at a 10,000 rpm and temperature of 25 °C. After remotion of supernatant, the solid was resuspended in 500 mL of distilled water, and another 0.32 g of LiCl was added under constant stirring for 24 h. After that, the solution was centrifuged again, following the same procedure as before. The resulting solid was resuspended for the third time in 500 mL of water, and another 0.32 g of LiCl was added, followed by further 24 h stirring. The clay was dialyzed in deionized water and purified using the Easypure^®^ RoDi until the negative test for chloride ions using 0.1 mol/L AgNO_3_ solution. After the lyophilization, the clay had cotton-like color and consistency.

Alg-based NPEs were prepared by the solution intercalation method. For this, 0.6 g of glycerol (Synth, Diadema-SP, Brazil) and 10 mL of deionized water were mixed in 100 mL beaker under constant stirring and heating at 80 °C. Next, 0.6 g of sodium alginate (Sigma, Cotia-SP, Brazil lot 41K0259) was gradually added together with an increasing amount of deionized water, while the temperature was raised to 160 °C, until a complete dissolution of Alg. After and still under continuous stirring, 0.4 g of LiClO_4_ (Sigma, Cotia-SP, Brazil) was solubilized, then was added 0.006–0.120 g of clay in 20 mL of deionized water (Table 1). Next, 0.5 g of formaldehyde (Synth, Diadema-SP, Brazil) was put into the Alg-based NPE solution and left under electromagnetic stirring for 24 h. Finally, the resulting solution was spilled into a Petri glass dish and dried at 40 °C for 24 h, resulting in membranes with thicknesses varying from 0.04 to 0.10 mm, as shown in Table 1.

The nanocomposites were characterized by X-ray diffraction using a Rigaku Rotaflex RU-200B with CuKα radiation (λ = 0.154 nm) and operating parameters of 50 kV and 100 mA. The 2θ varied from 5–80°.

Transmittance and reflectance were collected from Jasco V770 spectrophotometer in the wave range of 200–800 nm. Reflectance measurements were made using an integrating sphere. 

ATR–FTIR analyses were performed in Perkin Elmer Frontier spectrometer in the wavenumber from 600 to 4000 cm^−1^. The spectra are shown in Figures S1–S3 of Supplementary Material.

Impedance spectroscopy was used to evaluate the ionic conductivity of the Alg-based NPEs. The samples were pressed between stainless steel electrodes, under reduced pressure, in a homemade Teflon holder that was placed in a controlled furnace EDG 5P. Impedance data were collected with a Solartron SI 1260 operating in a frequency range 10^6^–10^−1^ Hz and AC amplitude of 5 mV.

## 4. Conclusions

Nanocomposite polymer electrolytes (NPEs), composed of sodium alginate (Alg) and modified montmorillonite SCa-3-Na^+^/SCa-3-Li^+^ clays, were synthesized and characterized. The X-ray analyses of the membrane samples evidenced the change of exfoliated to intercalated structures with the increase of clay quantity. The UV–Vis spectra showed that the transparency of the membranes decreased and reflectance increased with the increase of the clay content. The samples with 1–20 wt% of clay reached 76–87% of transmittance at 800 nm. On the other hand, the reflectance values attained their maxima of 11% at 320 nm for both Alg-based NPEs with 20 wt% of SCa-3-Na^+^ and SCa-3-Li^+^. All the membranes were tested for their ionic conductivities, and the best values of 2.77 × 10^−3^ at 25 °C and 1.96 × 10^−2^ S/cm^2^ at 70 °C were registered for Alg-based NPEs with 5 wt% SCa-3-Na^+^, and 1.22 × 10^−3^ S/cm^2^ at 25 °C and 1.30 × 10^−2^ S/cm^2^ at 70 °C for Alg-based NPEs with 10 wt% SCa-3-Li^+^. All samples displayed mostly linear dependence of log of the ionic conductivity as a function of the inverse of temperature, suggesting an Arrhenius (hopping) model for the ionic conduction. In summary, all the collected results have shown a positive influence of clay on the conductive properties of NPEs, which make them good electrolytes for electrochemical devices.

## Figures and Tables

**Table 1:** Alg-based NPEs clay content and membranes’ thicknesses.

Clay (wt%)	Clay Mass (g) Dissolved in 20 mL of H_2_O	Alg-Based NPE with Clay Thickness (mm)	
		SCa-3-Na^+^	SCa-3-Li^+^
0	0.000	0.04	0.04
1	0.006	0.05	0.09
5	0.030	0.06	0.08
10	0.060	0.06	0.05
15	0.090	0.07	0.06
20	0.120	0.10	0.08

**Table 2:** Values of ionic conductivity (σ) and activation energy (Ea) of Alg-based NPEs with 0–20% of SCa-3-Na^+^ and SCa-3-Li^+^.

Alg-Based NPE with Clay (wt%)		σ (S/cm^2^) at 25 °C	σ (S/cm^2^) at 70 °C	Ea (eV)
SCa-3-Na^+^	0	3.57 × 10^−4^	5.90 × 10^−3^	0.23
	1	1.34 × 10^−3^	1.36 × 10^−2^	0.19
	5	2.77 × 10^−3^	1.96 × 10^−2^	0.16
	10	2.00 × 10^−3^	1.53 × 10^−2^	0.18
	15	8.73 × 10^−4^	1.36 × 10^−2^	0.22
	20	1.84 × 10^−3^	1.36 × 10^−2^	0.18
SCa-3-Li^+^	1	5.36 × 10^−4^	9.02 × 10^−3^	0.23
	5	6.61 × 10^−4^	5.80 × 10^−3^	0.19
	10	1.22 × 10^−3^	1.30 × 10^−2^	0.23
	15	5.13 × 10^−4^	1.00 × 10^−2^	0.24
	20	4.62 × 10^−4^	6.52 × 10^−3^	0.22

## Supplementary Materials

The following are available online. Figure S1: FTIR spectra for Alg-based NPEs with 0–20 wt% of (a) SCa-3-Na^+^ and (b) SCa-3-Li^+^ clays. Figure S2: FTIR spectra for Alg-based NPEs with 0–20 wt% of SCa-3-Na^+^ in (a) 4000–2000, (b) 1750–1250, and (c) 1200–800 cm^−1^ wavenumber range. Figure S3: FTIR spectra for Alg-based NPEs with 0–20 wt% of SCa-3-Li^+^ in (a) 4000–2000, (b) 1750–1250, and (c) 1200–800 cm^−1^ wavenumber range.
