# Supplementary material for: Nanocomposite Polymer Electrolytes of Sodium Alginate and Montmorillonite Clay
Source: Molecules. 2021 Apr 8;26(8):2139. doi: 10.3390/molecules26082139 (PMC8068159; doi:10.3390/molecules26082139)
Supplement: Supplementary file 1 [file molecules-26-02139-s001.pdf]

Supplementary Material

The FTIR spectra of Alg-based NPEs without and with montmorillonite clays are shown in Figure S1. The NPE membrane without clay (0% of clay) exhibit almost the same characteristic bands as Alg-based NPEs with clay. The broad band at 3400 cm^{-1} resulted from the O-H stretching [1]. The stretching bands of C-H are at 2943 and 2886 cm^{-1}. The bands at 1620 and 1416 cm^{-1} are attributed to symmetric and asymmetric stretching vibrations of free sodium alginate carboxyl groups (COO^-) [2]. At 1080 cm^{-1} there is a stretching of C-O-C of the mannuronic and guluronic acid ring. This peak wavenumber changes depending of these two acids proportion in alginate sample [3].The band at 1032 cm^{-1} is controversial, Sugkugawa et al. [3] claims it comes from the O-H bending groups present in the polymer chain, but Helmiyati and Aprilliza [1] and Pathak et al. [2] assign it to C-O-C stretching. Alginate uronic acid functional groups usually are detected at around 924 cm^{-1} and mannuronic acid at around 852 cm^{-1} [1]. At the same wavenumbers, and probably overlapped, there are montmorillonite clay characteristic bands corresponding to Si-O-Si stretching at 993 cm^{-1}, Al-Al-OH deformation at 912 cm^{-1}, and coupled Al-O and Si-O out of plane at 617 cm^{-1} [4].

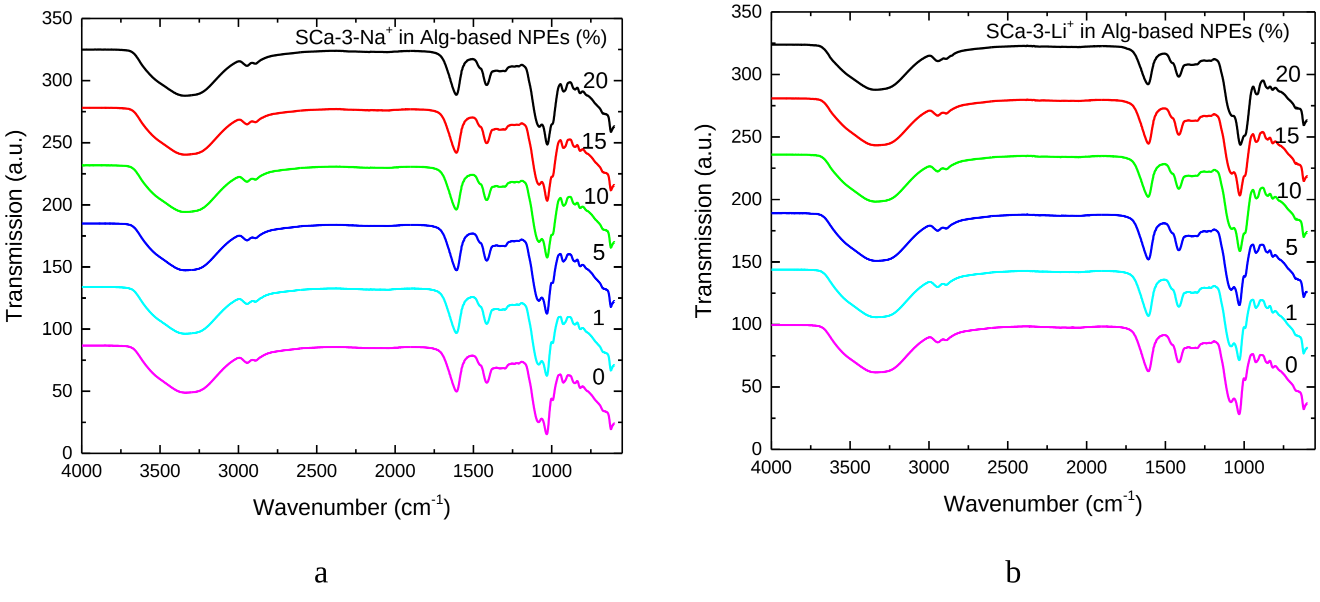

Figure S1. FTIR spectra for Alg-based NPEs with 0-20 wt% of (a) SCa-3-Na^+ and (b) SCa-3-Li^+ clays.

Closer analysis of these results (Figure S2) reveals that almost all the bands remain in the same position, except for the band as 1032 cm^{-1} that slightly shifts to lower wavenumbers after addition of clay. It reaches 1028 cm^{-1} for the sample with 20 wt.% of SCa-3-Na^+ (Figure S2c). Similar behavior was observed for the samples with SCa-3Li^+ (Figure S3) where this band shifted to 1024 cm^{-1} (Figure S3c). This can be due to the clay or simply measurement error.

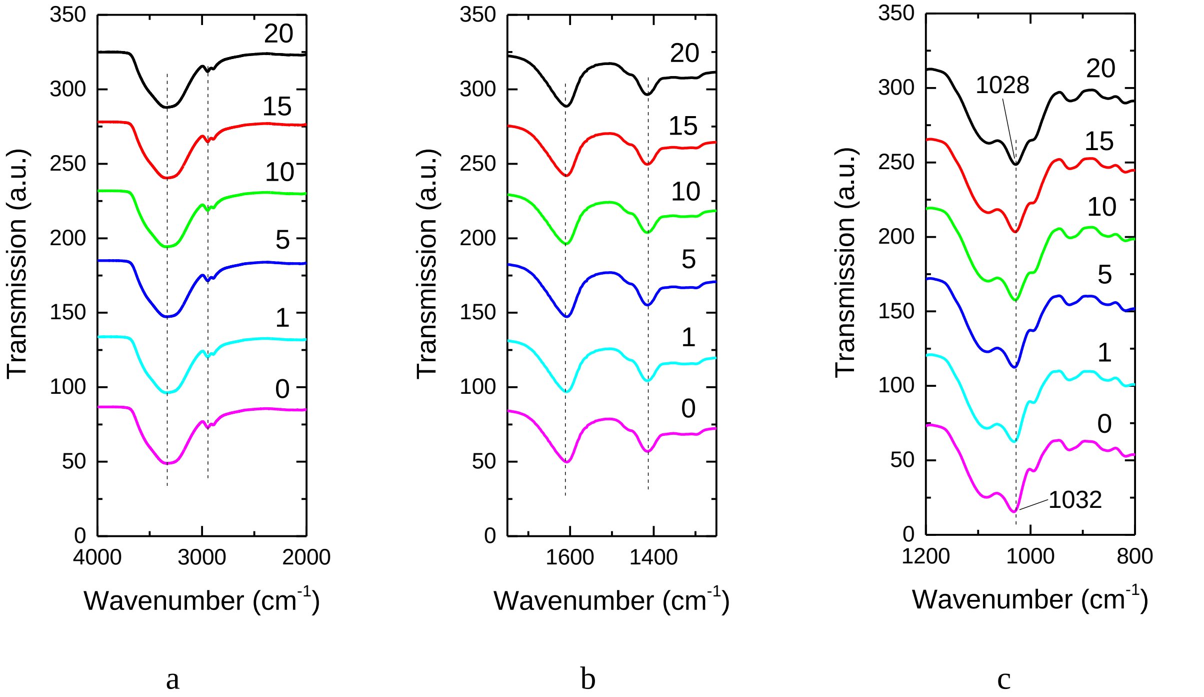

Figure S2. FTIR spectra for Alg-based NPEs with 0-20 wt% of SCa-3-Na^{+} in(a) 4000-2000, (b) 1750-1250, and (c) 1200-800 cm^{-1} wavenumber range.

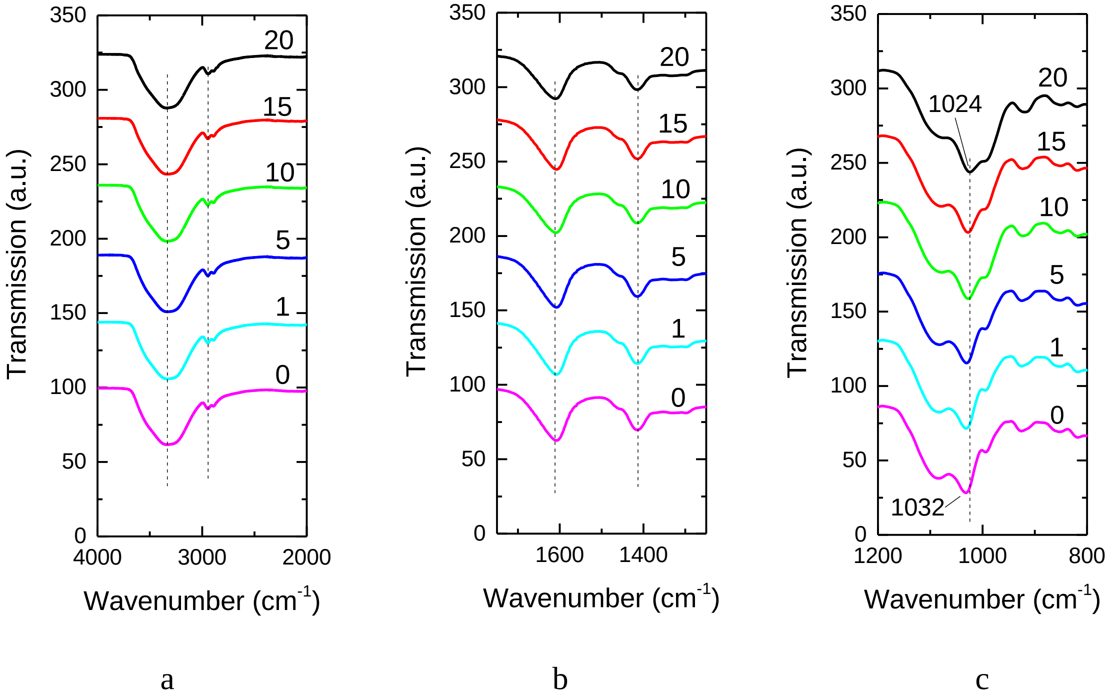

Figure S3. FTIR spectra for Alg-based NPEs with 0-20 wt% of SCa-3-Li^+ in (a) 4000-2000, (b) 1750-1250, and (c) 1200-800 cm^{-1} wavenumber range.

References

1. Aprilliza, M. Characterization and properties of sodium alginate from brown algae used as an ecofriendly superabsorbent. IOP Publ. 2017, 188, 012019.
2. Pathak, T.S.; San Kim, J.; Lee, S.-J.; Baek, D.-J.; Paeng, K.-J. Preparation of alginic acid and metal alginate from algae and their comparative study. J. Polym. Environ. 2008, 16, 198–204.
3. Sakugawa, K.; Ikeda, A.; Takemura, A.; Ono, H. Simplified method for estimation of composition of alginates by FTIR. J. Appl. Polym. Sci. 2004, 93, 1372–1377.
4. Sentanin, F.; Sabadini, R.; Barros, S.; Caliman, W.; Cavalheiro, C.; Kanicki, J.; Donoso, J.P.; Magon, C.J.; Silva, I.; Silva, M. Study of ionically conducting nanocomposites for reflective electrochromic devices. Electrochim. Acta 2019, 301, 174–182, doi:10.1016/j.electacta.2019.01.130.
